# Establishment and comparative analysis of *Agrobacterium*-mediated genetic transformation systems for *Actinidia valvata* and *Actinidia chinensis*

**DOI:** 10.3389/fpls.2026.1764453

**Published:** 2026-02-02

**Authors:** Junyao Xia, Jinfang Shu, Su Lin, Junjie Zhu, Fangbin Lin, Runxuan Fang, Liuqing Huo, Jinhua Liu, Kai Xu, Haijie Ma

**Affiliations:** 1 Key Laboratory of Quality and Safety Control for Subtropical Fruit and Vegetable, Ministry of Agriculture and Rural Affairs, Collaborative Innovation Center for Efficient and Green Production of Agriculture in Mountainous Areas of Zhejiang Province, College of Horticulture Science, Zhejiang Agriculture and Forestry (A&F) University, Hangzhou, Zhejiang, China; 2 TANG Co., Ltd., Hangzhou, Zhejiang, China

**Keywords:** Actinidia, Agrobacterium, genetic transformation, hairy root, high-pressure propagation-based transformation, plant regeneration

## Abstract

Kiwifruit (*Actinidia* spp.) is an economically important horticultural crop, yet functional genomics and molecular breeding efforts remain limited by low transformation efficiency, particularly in recalcitrant genotypes. In this study, *Agrobacterium*-mediated transformation systems were established and systematically optimized for *Actinidia chinensis* and *Actinidia valvata*. The optimized *Agrobacterium tumefaciens* GV3101 system achieved a 24% transformation efficiency in *A. chinensis* leaf explants, enabling complete plant regeneration within five months. In contrast, *A. valvata* showed only 4% efficiency, and its transformed call failed to initiate shoot organogenesis. To overcome this limitation, *Agrobacterium rhizogenes* K599-based methods were developed. The tissue culture system induced transgenic hairy roots at a rate of 68.7% without exogenous hormones, while a tissue culture-free protocol based on high-pressure propagation of woody stems reduced the transformation cycle to 4–5 weeks with 50% efficiency. Stable transgene integration and expression were confirmed by GFP fluorescence, PCR, and sequencing. Collectively, these transformation systems provide rapid, efficient, and reproducible tools that will substantially advance gene functional studies and molecular breeding in different kiwifruit species.

## 1 Introduction

Kiwifruit (*Actinidia* spp.) is a perennial deciduous vine valued worldwide for its remarkable nutritional composition and economic importance. The fruit is exceptionally rich in vitamin C (65.5–165 mg per 100 g fresh weight), greatly exceeding that of most common fruits, and also contains abundant dietary fiber, flavonoids, and actinidine, which contribute to its antioxidant capacity and metabolic health benefits (Rakha et al., 2025; Wang et al., 2021; Zhang et al., 2020). With the growing global demand, kiwifruit has become one of the most important subtropical fruit crops, particularly in China, where it represents a key component of the horticultural industry (Gao et al., 2022; Tutberidze et al., 2025). Extensive phenotypic and genetic diversity exists across *Actinidia* germplasms, including differences in fruit size, color, texture, and tolerance to various biotic and abiotic stresses. For example, the strong cold tolerance of *Actinidia arguta* and the attractive but disease-prone golden-fleshed cultivars (Ferrante et al., 2015; Sun et al., 2020). Understanding the molecular mechanisms and regulatory networks underlying these traits, such as vitamin C biosynthesis and stress response pathways, is fundamental for the genetic improvement and molecular breeding of high-quality, stress-resilient kiwifruit cultivars (Li et al., 2015; Liu et al., 2023; Nazir et al., 2024; Wang et al., 2023).

Over the past decades, the advancement of plant biotechnology has revolutionized crop breeding and functional genomics. Among the available techniques, *Agrobacterium*-mediated genetic transformation remains the most widely employed and reliable method due to its ability to transfer T-DNA into plant genomes with high stability and low copy number (Mohana et al., 2025; Pan et al., 2025; Tiwari et al., 2022). Nevertheless, several alternative strategies have also been explored to expand transformation applicability across diverse plant taxa. These include *Agrobacterium rhizogenes*-mediated transformation for hairy root induction (Lin et al., 2025; Liu et al., 2024; Lu et al., 2025), polyethylene glycol (PEG)-mediated protoplast transformation (Hong et al., 2023; Lim et al., 2021; Qi et al., 2025), biolistic particle delivery (Miller et al., 2021; Poddar et al., 2023), and emerging nanoparticle-based delivery systems (Islam et al., 2024; Mittal et al., 2025; Yong et al., 2024). PEG-mediated transformation is particularly useful for transient gene expression assays, such as promoter activity and subcellular localization analyses, though the limited regeneration ability of protoplasts hampers its utility for stable transformation (Gao et al., 2025). Biolistic approaches overcome host range limitations but often result in tissue damage and complex multi-copy insertions, causing transgene silencing and instability (Miller et al., 2022; Thorpe et al., 2025). Nanotechnology-based systems promise low-damage, high-efficiency gene delivery but remain at a developmental stage lacking standardized protocols (Shivashakarappa et al., 2025). Despite these innovations, establishing efficient and reproducible transformation systems for woody fruit crops such as kiwifruit remains a formidable challenge due to their long growth cycles, high lignification, and abundance of phenolic compounds that hinder bacterial infection and regeneration.

In woody perennials, *Agrobacterium tumefaciens*-mediated transformation has been widely applied to crops such as apple, pear, citrus, and litchi through species-specific optimization of explant selection, co-cultivation conditions, and hormone balance (Qin et al., 2025; Xue et al., 2023; Yang et al., 2025; Zhang et al., 2025). For example, using this method, *Malus domestica* (apple) and *Actinidia chinensis* (kiwifruit) produced transgenic roots in 3 weeks, with *MdWOX5* increasing the regeneration efficiency to 20.6%; in *Malus domestica* ‘Gala’, knocking out *MdPDS* yielded albino mutants, and overexpressing *MdARF5* enhanced somatic embryo proliferation and regeneration efficiency (Liu et al., 2024; Yang et al., 2025). Similar strategies in cereals and vegetables have accelerated breeding cycles for stress-resistant and high-yield varieties (Castellanos-Arévalo et al., 2020; Haroon et al., 2022; Mei et al., 2024). However, in *Actinidia* species, transformation remains highly genotype-dependent. Although successful transgenic plants have been obtained in *A. chinensis*, transformation efficiencies are generally low and inconsistent across genotypes (Li et al., 2024; Wang and Liu, 2024). The recalcitrance of many kiwifruit species, such as *A. valvata*, is largely attributable to inefficient *Agrobacterium*-host interactions, secretion of phenolic inhibitors, and difficulty in plant regeneration during tissue culture (Wang et al., 2021; Wu et al., 2020). Consequently, the absence of robust transformation platforms has severely limited functional genomics research and precise gene manipulation in kiwifruit breeding.

*A. rhizogenes*, which naturally induces hairy root formation via its Ri plasmid-encoded *rol* gene cluster, provides an alternative route for genetic transformation, particularly suited for root biology and gene functional studies (Kumar et al., 2025). This bacterium can trigger organogenesis without exogenous hormones, enabling more rapid and efficient transgene introduction into recalcitrant species. Previous studies in apple and soybean have demonstrated that *A. rhizogenes*-mediated systems can significantly reduce transformation time while maintaining high transgene stability (Huang et al., 2022; Liu et al., 2024). Recently, the integration of tissue culture-free approaches, such as high-pressure propagation and direct stem infection, has further simplified transformation workflows, offering potential for scalable applications in woody crops. However, these methods have not been systematically tested or compared in *Actinidia* species.

The comparative evaluation of *A. tumefaciens* and *A. rhizogenes*-mediated systems thus represents a pivotal step toward understanding genotype-specific constraints and developing more efficient transformation protocols for kiwifruit. Optimizing key parameters such as bacterial strain, explant type, infection time, co-cultivation duration, and regeneration conditions is crucial to improve compatibility across genotypes. Additionally, implementing dual verification systems, combining GFP fluorescence imaging with molecular validation through PCR and sequencing, ensures accurate identification of true transgenic events while minimizing false positives from bacterial residues or chimerism. These methodological refinements can not only enhance transformation success rates but also provide standardized frameworks for reproducible genetic manipulation in kiwifruit and other woody crops.

To address the limited availability of efficient transformation systems in kiwifruit, this study was designed to develop practical *Agrobacterium*-mediated approaches suitable for different *Actinidia* genotypes. The work involved establishing transformation procedures using *A. tumefaciens* GV3101 and *A. rhizogenes* K599, with systematic optimization of key steps including explant preparation, agro-infiltration, co-cultivation, and selection. In addition, a tissue culture-free transformation strategy based on high-pressure propagation of woody stems was designed to explore a simplified route for gene introduction under non-aseptic conditions. The overall goal of the study is to construct reliable and reproducible transformation protocols that can support gene function analysis and provide technical references for molecular breeding in kiwifruit.

## 2 Materials and methods

### 2.1 Plant materials and growth conditions

*A. chinensis* ‘Qihong’ (QH) and *A. valvata* ‘Duie’ (DE) cultured in MS medium (Murashige and Skoog basal medium containing 30 g L^-1^ sucrose and 8 g L^-1^ agar, adjusted to pH 5.8 before autoclaving.) were used as experimental materials. The plantlets were maintained in a growth chamber under controlled conditions: temperature 24-26 °C, photoperiod 16-h light/8-h dark, and light intensity of approximately 2500 lx. Subculturing was performed every four weeks to maintain vigorous growth and ensure material consistency. Healthy, uniformly developed shoots were selected as explant sources for subsequent transformation experiments. Both materials mentioned above originated from the Kiwifruit Germplasm Repository of the College of Horticulture Science, Zhejiang A&F University, Lin’an District, Hangzhou City, Zhejiang Province.

### 2.2 Bacterial strains

Two *Agrobacterium* strains were used in this study: *A. tumefaciens* GV3101 and *A. rhizogenes* K599. Both strains were obtained from Weidi Biotechnology Co., Ltd. (Shanghai, China). Each strain was stored as 50% glycerol stocks at -80 °C and revived on selective solid media prior to use. GV3101 was cultured on yeast extract peptone (YEP) medium, while K599 was cultured on tryptone yeast medium. Antibiotics were added to maintain plasmid selection: kanamycin (50 mg L^-1^) and rifampicin (50 mg L^-1^) for GV3101, and kanamycin (50 mg L^-1^) and streptomycin (50 mg L^-1^) for K599. Single colonies were inoculated into liquid medium of the same composition and grown at 28 °C with shaking at 200 rpm for 12 h before being used for transformation assays.

### 2.3 Plasmids and antibiotics

The binary vector 1380-GFP was used for all transformation experiments. This plasmid carries the GFP (green fluorescent protein) encoding gene under the control of a constitutive promoter and a kanamycin resistance gene for selection of transgenic tissues. The plasmid was introduced into *A. tumefaciens* GV3101 and *A. rhizogenes* K599 by the freeze-thaw method and verified by colony PCR prior to use.

Antibiotics used in this study included kanamycin sulfate, streptomycin sulfate, and rifampicin, all purchased from Sangon Biotech Co., Ltd. (Shanghai, China). In plant tissue culture, kanamycin was used as a selective agent at concentrations of 100 mg L^-1^ for shoot induction medium and 50–100 mg L^-1^ for callus selection, while cefotaxime (cef, 100–400 mg L^-1^) was applied to eliminate residual *Agrobacterium* following co-cultivation.

### 2.4 Chemical reagents and media

All chemical reagents used in this study were of analytical grade and purchased from Sangon Biotech Co., Ltd. (Shanghai, China). Key reagents included MgCl_2_ (CAS: 7786-30-3), NaCl (CAS: 7647-14-5), NaOH (CAS: 1310-73-2), CaCl_2_ (CAS: 10043-52-4), DMSO (CAS: 67-68-5), citric acid (CAS: 77-92-9), betaine (CAS: 107-43-7), acetosyringone (AS, CAS: 2478-38-8), CTAB (CAS: 57-09-0), MES (CAS: 4432-31-9), 6-benzylaminopurine (6-BA, CAS: 1214-39-7), naphthaleneacetic acid (NAA, CAS: 86-87-3), indole-3-butyric acid (IBA, CAS: 133-32-4), indole-3-acetic acid (IAA, CAS: 87-51-4), and trans-zeatin (TZ, CAS: 1637-39-4).

The following media were prepared and used during bacterial culture and plant transformation: TY Solid Medium: 3 g/L Yeast extract, 5 g/L Tryptone, 10 mM CaCl_2_, 15 g/L Agar. TY Liquid Medium: 3 g/L Yeast extract, 5 g/L Tryptone, 10 mM CaCl_2_. YEP Solid Medium: 10 g/L Yeast extract, 10 g/L Tryptone, 5 g/L NaCl, 15 g/L Agar. YEP Liquid Medium: 10 g/L Yeast extract, 10 g/L Tryptone, 5 g/L NaCl. MS Basal Medium: 4.43 g/L MS salts, 30 g/L Sucrose, 8 g/L Agar, pH 5.8. Shoot Induction Medium: MS Basal Medium supplemented with 100 mg/L cef, 100 mg/L kanamycin, 3 mg/L TZ, and 0.5 mg/L IAA, pH 5.8. Co-cultivation Medium: MS Basal Medium supplemented with 100 μM AS, pH 5.8. Root Induction Medium: MS Basal Medium supplemented with 400 mg/L cef, 0.6 mg/L IBA, pH 5.8. MES Infiltration Buffer: 10 mM MgCl_2_, 10 mM MES, 100 μM AS, pH 5.8. MS Infiltration Buffer: 4.43 g/L MS salts, 20 g/L Sucrose, 100 μM AS, 5.88 g/L Citric Acid, 0.5 mg/L Betaine, pH 5.2. Citric acid was included to stabilize pH during infiltration, while betaine served as an osmoticum to enhance bacterial viability and T-DNA delivery.

For nucleic acid extraction, the CTAB buffer consisted of 4 g CTAB, 16.364 g NaCl, 20 mL 1 M Tris–HCl (pH 8.0), and 8 mL 0.5 M EDTA, with the final volume adjusted to 200 mL using distilled water.

Stock solutions of plant growth regulators (6-BA, NAA, IBA, IAA, and TZ) were prepared at 2 mg mL^-1^ by dissolving in a small amount of 1 M NaOH and diluting with sterile water. Acetosyringone stock (50 mg mL^-1^) was prepared in DMSO. All stock solutions were sterilized by filtration (0.22 μm) and stored at -20 °C until use.

### 2.5 GV3101-mediated genetic transformation of kiwifruit

The GV3101, harboring the binary vector 1380-GFP, was employed to establish transformation systems for both *A. valvata* and *A. chinensis* tissue-cultured plantlets. The transformation procedure included bacterial activation, preparation of infiltration suspension, explant infiltration, co-cultivation, and selective regeneration.

Bacterial activation and preparation of infiltration suspension: GV3101 stored at -80 °C was streaked on YEP solid medium and incubated at 28 °C for 48 h. A single colony was inoculated into 800 μL YEP liquid medium containing 50 mg/L kanamycin and 50 mg/L rifampicin, followed by shaking incubation at 28 °C and 200 rpm for 12 h. The bacterial culture was expanded to 100 mL under the same conditions until the optical density reached OD_600_ = 0.8-1.2. Cells were collected by centrifugation (5000 × g, 10 min) and resuspended in MS infiltration buffer to a final OD_600_ = 0.8 for subsequent infection.

Explant preparation and agro-infiltration: Healthy, fully expanded leaves from *in vitro A. chinensis* plantlets were excised into 1 × 1 cm segments and used as explants. The explants were immersed in the prepared bacterial suspension for 25 min with gentle agitation to ensure even contact and bacterial infiltration. After infiltration, the explants were blotted on sterile filter paper to remove excess bacterial solution and reduce contamination during co-cultivation. Co-cultivation: The infiltrated explants were transferred onto co-cultivation medium and incubated in darkness at 26 °C for 2 days to facilitate T-DNA transfer. After co-cultivation, explants were rinsed several times with sterile water containing 200 mg/L cefotaxime to remove residual *Agrobacterium*, blotted dry, and transferred to the selection medium.

Selection and regeneration: Explants were cultured on shoot induction medium containing the appropriate antibiotics and plant growth regulators, under controlled environmental conditions of 24-26 °C, 16-h light/8-h dark photoperiod, and subcultured every 4 weeks. Developing callus tissues from wound sites were maintained on fresh selection medium for continuous proliferation and subsequent shoot induction. Emerging shoots were excised and transferred onto root induction medium to promote root formation under the same environmental conditions.

### 2.6 K599-mediated genetic transformation of kiwifruit

The K599, harboring the binary vector 1380-GFP, was used for the genetic transformation of *A. valvata* tissue-cultured plantlets. The transformation process included bacterial activation, preparation of infiltration suspension, explant infiltration, co-cultivation, and selective regeneration culture.

Bacterial activation and preparation of infiltration suspension: K599 was retrieved from -80 °C glycerol stock and streaked onto TY solid medium, followed by incubation in darkness at 28 °C for 48 h. A single colony was inoculated into 800 μL TY liquid medium containing 50 mg/L kanamycin and 50 mg/L streptomycin, and cultured at 28 °C and 200 rpm for 12 h. The bacterial culture was expanded to 100 mL under the same conditions until OD_600_ reached 0.8-1.2. Cells were collected by centrifugation (5000 × g, 10 min) and resuspended in MES infiltration buffer to an OD_600_ = 0.8 for infiltration. The bacterial suspension was then incubated in the dark at room temperature for 2–3 h to induce virulence gene expression prior to plant tissue infiltration.

Explant preparation and infiltration: Healthy, uniformly growing *A. valvata* tissue-cultured plantlets were selected as explant sources. Fully expanded young leaves were excised into 1 × 1 cm segments and immersed in the prepared bacterial suspension for 25 min with gentle shaking to allow bacterial adhesion and T-DNA transfer. After infiltration, the explants were blotted on sterile filter paper to remove excess bacterial liquid and prevent overgrowth during co-cultivation.

Co-cultivation: The infiltrated explants were transferred onto co-cultivation medium and incubated in darkness at 26 °C for 2 days. Following co-cultivation, explants were rinsed several times with sterile water containing 200 mg/L cefotaxime, blotted dry on sterile filter paper, and transferred to selective medium for further culture.

Regeneration: Explants were cultured on MS medium containing kanamycin, cefotaxime, and plant growth regulators (0.6 mg/L IBA) for 14 weeks, after which they were transferred to hormone-free MS medium supplemented only with kanamycin and cefotaxime. Cultures were maintained at 24-26 °C under a 16-h light/8-h dark photoperiod and subcultured every 4 weeks. Under K599-mediated transformation, the *rol* gene cluster in the Ri plasmid endogenously induces root primordia formation without the need for exogenous hormones. Consequently, the induction of transgenic hairy roots occurred directly on the selection medium, where callus proliferation and root differentiation were allowed to proceed simultaneously until distinct hairy roots developed from the wound sites.

### 2.7 High-pressure propagation-mediated genetic transformation of *A. valvata* using K599

A high-pressure propagation-mediated, tissue culture-free transformation system was established for *A. valvata* using the K599 carrying the 1380-GFP vector. In this method, infiltration and induction were performed directly on the branches of intact plants under greenhouse conditions, enabling *in planta* transformation without the need for sterile tissue culture.

Preparation of bacterial suspension: The preparation of the K599 bacterial suspension was conducted as described above in Section 2.6, using MES infiltration buffer and adjusting the bacterial concentration to OD_600_ = 0.8 prior to infiltration. Agro-infiltration of intact plants: Healthy *A. valvata* plants with a stem diameter of 5–9 mm was selected as recipients. On each target branch, longitudinal wounds (approximately 1.0-1.5 cm in length, penetrating to the cambium without damaging the pith) were made using a sterile scalpel. Sterile filter paper strips (1.5 × 3 cm) were soaked in the bacterial suspension and tightly wrapped around the wounded area for 20 min in the dark to facilitate bacterial infiltration and T-DNA delivery. After treatment, the filter paper was removed, and excess bacterial solution on the wound surface was carefully blotted with sterile absorbent paper.

Immediately after infiltration, the treated wound region was enclosed with a black rooting box (polyethylene, approximately 5 × 5 × 8 cm, with side ventilation slits for gas exchange) filled with sterile substrate (vermiculite:matrix = 1:3, particle size 2–4 mm, autoclaved at 121 °C for 30 min). The rooting box was securely fixed to the stem to maintain contact between the infected site and the substrate. The entire plant was cultivated under greenhouse conditions at 24-26 °C, 70-80% relative humidity, and a 16-h light/8-h dark photoperiod. The substrate was kept moist but not waterlogged, and the system was monitored regularly to maintain stable humidity and temperature.

Induction of transgenic tissues: The *rol* genes on the Ri plasmid of K599 induced cell dedifferentiation and root primordia formation at the infiltrated wound sites without the addition of exogenous hormones. Under greenhouse propagation conditions, transgenic callus and hairy roots gradually developed from the cambial region at the infected sites. Samples of these tissues were collected for molecular and fluorescence analysis once sufficient growth was observed.

### 2.8 GFP fluorescence detection in transgenic kiwifruit shoots

Transgenic shoots (experimental group) and wild-type shoots (control group) were thoroughly washed with sterile water. Leaf samples were transversely and longitudinally sectioned using a blade. The sections were observed and imaged using a confocal laser scanning microscope (OLYMPUS FV3000). The excitation wavelength was set at 488 nm, and emission was collected between 505–550 nm. Images were captured using a 10× objective lens. Calculate the genetic transformation efficiency of both kiwifruit species using the following formula: Genetic transformation efficiency = (Number of GFP-positive explants/Total number of explants infected with Agrobacterium) × 100%, in order to quantitatively evaluate the effectiveness of the transformation system. Transformation efficiency was calculated from three independent biological replicates, each consisting of 30–40 explants. Data are presented as mean ± standard deviation (SD).

### 2.9 Verification of transgenic kiwifruit

Genomic DNA was extracted from putative transgenic and wild-type tissues of *A. chinensis* and *A. valvata* using a modified CTAB method. Sample preparation and lysis: Fresh leaves or transgenic tissues (approximately 100 mg) were placed in 2 mL centrifuge tubes containing one sterile steel bead. Samples were flash-frozen in liquid nitrogen and ground to a fine powder using a tissue homogenizer (30 Hz, 60 s). Subsequently, 1.0 mL of pre-warmed (65 °C) CTAB extraction buffer (adjusted to pH 8.0 before use) was added, and samples were mixed thoroughly and incubated in a 65 °C water bath for 2 h to ensure complete cell lysis and nucleic acid release. Phase separation and DNA precipitation: After incubation, tubes were centrifuged at 12, 000 rpm for 10 min. Approximately 700 μL of the supernatant was transferred to a new tube, mixed with an equal volume of chloroform:isoamyl alcohol (24:1, v/v), and gently inverted several times. The mixture was centrifuged again at 12, 000 rpm for 10 min, and 600 μL of the upper aqueous phase was transferred to a new tube. An equal volume of isopropanol was added to precipitate DNA, and samples were incubated on ice (or at -20 °C) for 5–10 min. DNA was collected by centrifugation at 12, 000 rpm for 10 min, washed with 1 mL of 70% ethanol, and centrifuged again for 5 min. The supernatant was discarded, and the DNA pellet was air-dried until translucent. Finally, the DNA was dissolved in 30 μL of nuclease-free water (ddH_2_O) and stored at -20 °C until use. PCR amplification and verification: The extracted DNA was used as a template for PCR analysis to verify the presence of the *GFP* transgene. PCR reactions were performed using 2× Rapid Taq Master Mix (Vazyme, China) and the following primer pair specific to the *GFP* sequence: Forward primer (1380-GFP-F): 5′-TAGTTCATCCATGCCATGTGTA-3′; Reverse primer (1380-GFP-R): 5′-CTTCAAGAGCGCCATGCCTGA-3′. The PCR amplification was carried out in a total volume of 25 μL, including 12.5 μL of 2× Master Mix, 1 μL of each primer (10 μM), 2 μL of genomic DNA template, and 8.5 μL of ddH_2_O. The cycling conditions were as follows: initial denaturation at 95 °C for 3 min, followed by 35 cycles of denaturation at 95 °C for 15 s, annealing at 52 °C for 15 s, and extension at 72 °C for 15 s, with a final extension at 72 °C for 5 min. Amplification products were analyzed by 1% agarose gel electrophoresis (150 V, 150 mA, 25 min) and visualized under UV illumination. PCR-positive bands corresponding to the expected fragment size were further purified and sequenced to confirm the presence of the *GFP* transgene. The expected band size of PCR products was purified and sequenced. The obtained sequences were aligned with the reference *GFP* coding region using SnapGene software to verify sequence existence.

The expression of the *GFP* reporter gene in putative transgenic tissues of *A. chinensis* and *A. valvata* was examined using both handheld fluorescence illumination and confocal laser scanning microscopy. For preliminary screening, regenerated shoots, callus, and hairy roots were observed under a handheld blue-light excitation source equipped with a green emission filter in a dark environment. The appearance of bright green fluorescence at wound sites or throughout the tissues was recorded as a preliminary indicator of successful transformation, while wild-type tissues were used as negative controls. For detailed cellular-level analysis, fluorescence-positive samples were further examined using a confocal laser scanning microscope (OLYMPUS FV3000, Japan). Fresh leaf or root sections were washed with sterile water and mounted on glass slides. Imaging was performed at an excitation wavelength of 488 nm and an emission range of 505–550 nm, using identical settings for both transgenic and control samples. The presence of uniform green fluorescence within the cytoplasm of transgenic cells was used to confirm the functional expression of the *GFP* transgene.

## 3 Results

### 3.1 Establishment of *A. tumefaciens*-mediated genetic transformation systems for *A. valvata* and *A. chinensis*

**Figure 1:**
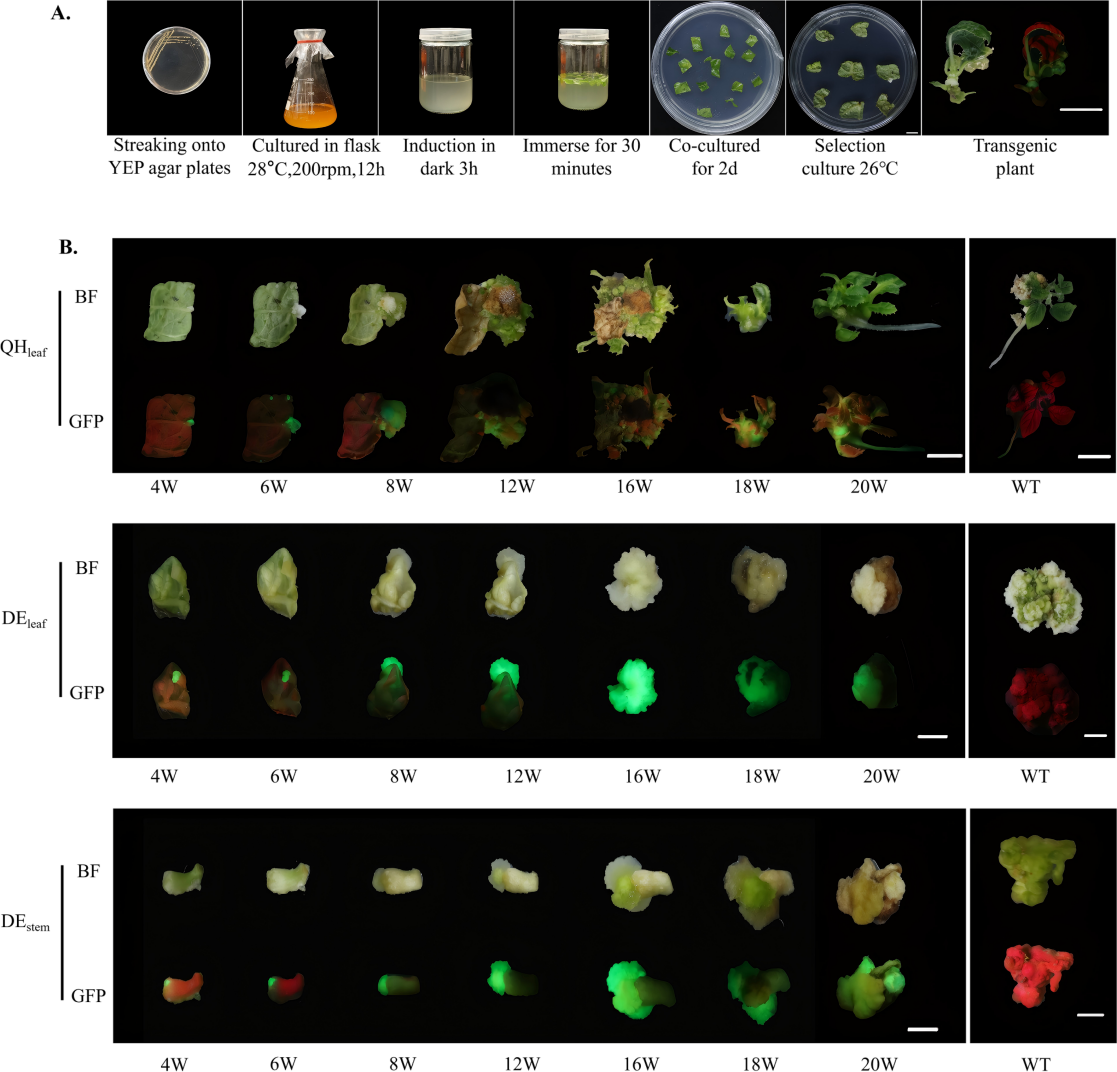
Establishment of *A. tumefaciens*-mediated genetic transformation systems for *A. chinensis* and *A. valvata*. **(A)** Schematic representation of the transformation procedure using *A. tumefaciens* GV3101, including bacterial activation, infiltration, co-cultivation, selection, and regeneration of transgenic plantlets. **(B)** Phenotypic progression of leaf explants from *A. chinensis* and *A. valvata* at various stages of transformation. GFP fluorescence images show successful transgene expression, with notable differences in organogenesis between the two species (‘QH’ represents *A. chinensis*, ‘DE’ represents *A. valvata*): in QH, GFP-positive callus developed into plantlets with shoot formation 18–20 weeks, while in DE, GFP-positive callus failed to initiate shoot organogenesis, remaining as proliferative callus. QH_leaf_, leaf of QH; DE_leaf_, leaf of DE; DE_stem_, stem of DE; BF, Bright-field; W, weeks; Scale bar = 1 cm.

To establish a stable and reproducible *A. tumefaciens*-mediated genetic transformation system for kiwifruit, *A. chinensis* and *A. valvata* were selected as representative genotypes. These two species differ significantly in regenerative ability and physiological properties, providing a suitable contrast for system development. Leaf discs from *in vitro*-grown shoots were infiltrated with GV3101 harboring the 1380-GFP binary vector. The entire process included bacterial activation, infiltration, co-cultivation, callus induction, and regeneration culture (**Figure 1A**). After infiltration and co-cultivation for 2 days, the explants were transferred to selection medium containing kanamycin and cefotaxime. Callus formation was observed at wound sites of both genotypes approximately four weeks after infiltration (**Figure 1B**). GFP fluorescence appeared in portions of the induced callus, indicating successful T-DNA delivery. In *A. chinensis*, the GFP-positive callus gradually developed into green, compact tissues, from which shoot primordia emerged after approximately 12 weeks of culture, followed by complete shoot regeneration at around 16 weeks and subsequent rooting within 20 weeks in selection medium (MS medium containing kanamycin, cef, 3mg L^-1^ TZ and 0.5 mg L^-1^ IAA) (**Figure 1B**). In contrast, *A. valvata* formed proliferative GFP-positive callus with strong growth potential but did not produce adventitious shoots during the same period (**Figure 1B**). These results demonstrate that the established GV3101-mediated system can achieve stable gene introduction and expression in both *A. chinensis* and *A. valvata*, though the morphogenic response differs between genotypes.

### 3.2 Verification of transgene integration and expression in transformed tissues

**Figure 2:**
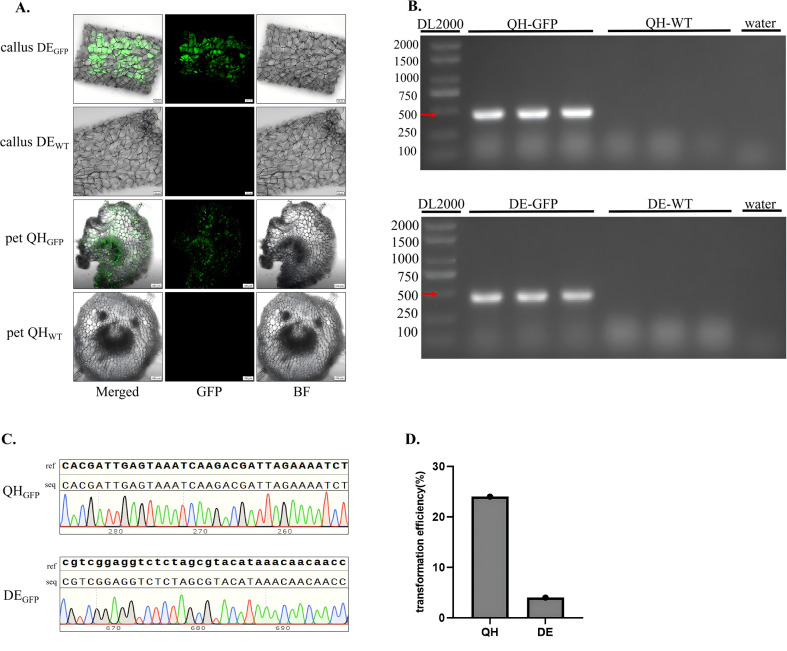
Phenotypic and molecular validation of transgenic kiwifruit plants. **(A)** Fluorescence microscopy images of transformed and control tissues (‘QH’ represents *A. chinensis*, ‘DE’ represents *A. valvata*). In transgenic lines (callus DE_GFP_ and pet QH_GFP_), strong green fluorescence is observed in the GFP channel, corresponding precisely with the tissue structure in merged and BF No GFP fluorescence is detected in controls (callus DE_WT_ and pet QH_WT_), confirming specific reporter expression in transgenic samples. Images were captured with a 10× objective. (callus DE_GFP:_ callus of DE_GFP_; pet QH_GFP:_ leaf of QH_GFP_; callus DE_WT:_ callus of DE_WT_; pet QH_WT:_ pet of QH_WT_; pet:Petiole). **(B)** PCR verification of the *GFP* gene. A single expected band (460 bp) is detected in QH_GFP_ and DE_GFP_ lanes using DL2000 as a DNA marker, verifying the presence of the transgene. No amplification is observed in QH_WT_, DE_WT_, or negative controls, confirming successful and specific transgene integration. **(C)** Sequences alignment of *GFP* amplicons from QH_GFP_ and DE_GFP_ samples. Clear, well-defined peaks and complete sequence alignment with the reference *GFP* sequence validate the accuracy and integrity of the integrated transgene. (ref: reference sequence; seq: sequenced PCR products). **(D)** Quantitative analysis of transformation efficiency. The **(A)***chinensis* genotype exhibits a significantly higher transformation efficiency (24%) compared with *A. valvata* (4%).

A dual verification system combining fluorescence-specific detection and molecular biological validation was employed to confirm the successful integration and expression of the *GFP* reporter gene in transformed kiwifruit tissues (**Figure 2**). This two-tier system effectively eliminated false positives caused by residual *Agrobacterium* cells or non-specific amplification, thereby ensuring the reliability of transformation results. For fluorescence-specific detection, sections of transgenic tissues from *A. chinensis* and *A. valvata* were examined under a confocal laser scanning microscope using wild-type tissues as negative controls. Imaging parameters were set precisely to distinguish GFP fluorescence from chlorophyll autofluorescence, excitation at 488 nm, emission at 505–550 nm for GFP, and a chlorophyll autofluorescence peak at approximately 685 nm. The results revealed that all cells of transgenic tissues exhibited bright and uniform green fluorescence, with no evidence of chimerism or mosaic expression (**Figure 2A**). In contrast, no GFP signal was detectable in wild-type tissues, confirming that the observed green fluorescence originated exclusively from functional GFP protein expression rather than background autofluorescence. For molecular biological validation, genomic DNA was extracted from both transgenic and wild-type leaves. PCR amplification with GFP-specific primers (Forward primer (1380-GFP-F): 5′-TAGTTCATCCATGCCATGTGTA-3′; Reverse primer (1380-GFP-R): 5′-CTTCAAGAGCGCCATGCCTGA-3′) produced a clear, single band at the expected size (460 bp) in transgenic samples, whereas no amplification was detected in wild-type controls (**Figure 2B**). The PCR-positive fragments were purified and subjected to Sanger sequencing, and alignment of the obtained sequences using SnapGene software showed 100% identity with the reference GFP coding sequence (**Figure 2C**). These results confirmed accurate and stable integration of the target gene into the kiwifruit genome and its functional expression at the cellular level. Quantitative evaluation based on GFP and molecular verification indicated a transformation efficiency of approximately 24% in *A. chinensis* and 4% in *A. valvata* (**Figure 2D**).

### 3.3 Establishment of *A. rhizogenes*-mediated genetic transformation system in *A. valvata*

**Figure 3:**
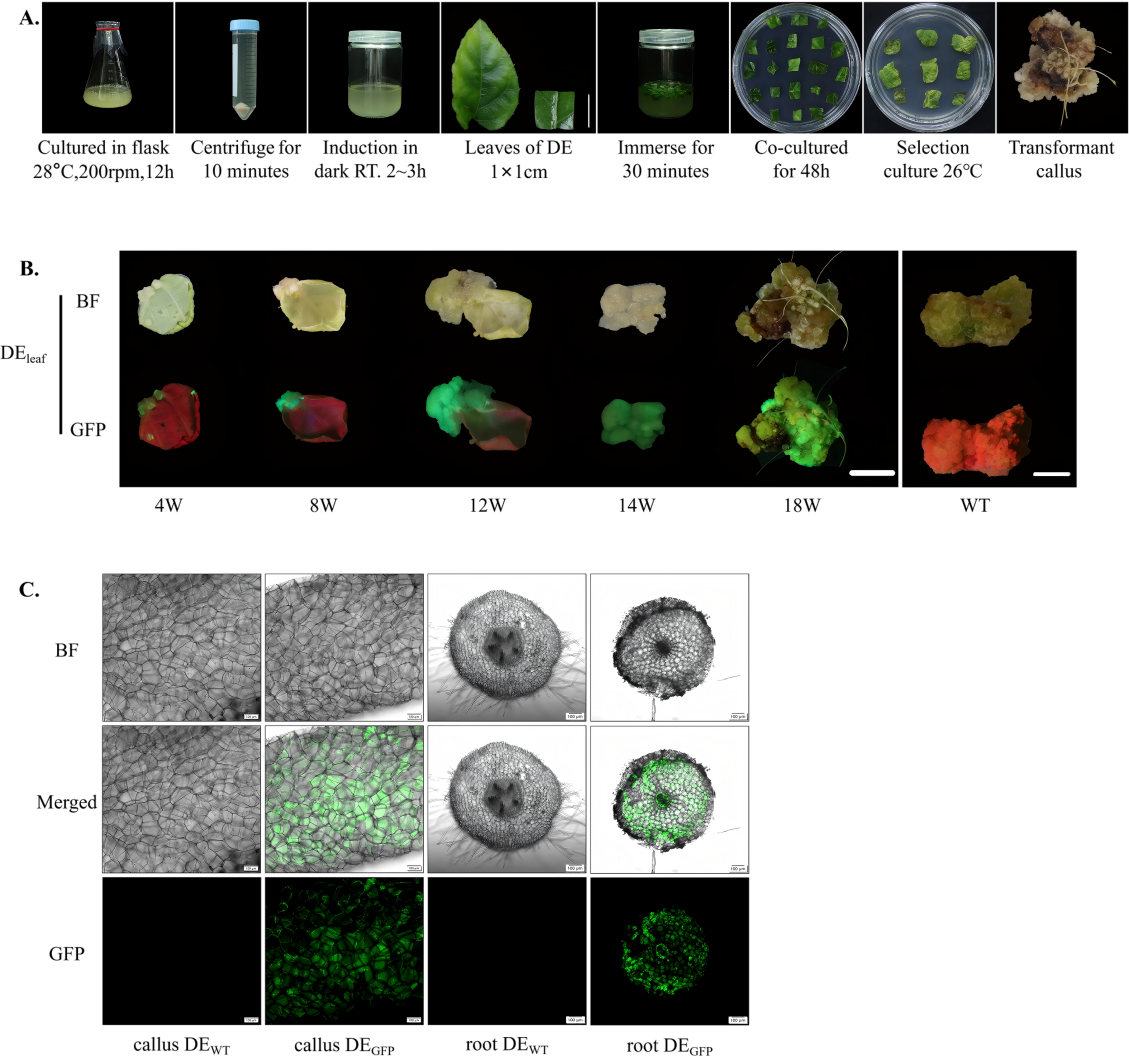
Establishment of **(A)** rhizogenes-mediated transformation system for A. valvata. **(A)** Schematic representation of the transformation workflow mediated by *A. rhizogenes* K599. The procedure includes bacterial culture at 28 °C and 200 rpm for 12 h, centrifuge for 10 min, induction under dark conditions at room temperature for 2–3 h, selection of *A. valvata* (DE) leaf discs (1 × 1 cm), immersion in bacterial suspension for 30 min, co-cultivation for 48 h, selective culture at 26 °C for screening of transformants; and subsequent acquisition of transformed callus. Scale bar = 1 cm. **(B)** Phenotypic comparison of the DE_leaf_ at different culture stages (4W, 8W, 12W, 14W, and 18W) and WT, showing morphological changes under BF and GFP fluorescence. Progressive tissue development and the corresponding variation in fluorescence intensity reflect transgene expression over time. Scale bar = 1 cm. **(C)** Observation of transgenic callus (callus DE_GFP_) and roots (root DE_GFP_) under BF, merged, and GFP channels. Distinct green fluorescence signals in transgenic samples confirm successful gene transfer and expression, while no fluorescence is detected in control of callus (callus DE_WT_) and roots (root DE_WT_). Images were captured with a 10× objective. (root DE_GFP:_ root of DE_GFP_; root DE_WT:_ root of DE_WT_).

To expand the available transformation systems for kiwifruit and assess the compatibility of *A. valvata* with *A. rhizogenes*, a tissue culture-based genetic transformation system mediated by strain K599 carrying the 1380-GFP vector was developed. This system complements the *A. tumefaciens*-mediated approach and provides an efficient route for studying root-specific gene function in *A. valvata*. In this system, *A. rhizogenes* K599 exhibited strong infectivity toward *A. valvata* explants. Following infiltration and co-cultivation, wound sites on *A. valvata* leaf explants developed visible callus after approximately 4 weeks, with a callus induction rate exceeding 80%. The callus enlarged progressively, displaying a friable texture and light-yellow color typical of active cell proliferation (**Figure 3A**). Comparative hormone regulation experiments demonstrated a clear influence of exogenous hormones on callus developmental fate. When explants were cultured on selection medium supplemented with the same cytokinin–auxin combinations used in the *A. tumefaciens*-mediated system, the transgenic callus continued to proliferate without organ differentiation, forming compact tissue masses (**Figure 3B**, 4–14 weeks). In contrast, when transferred to hormone-free MS medium, the same transgenic callus began to undergo spontaneous organogenic transition, producing numerous hairy roots from the transgenic callus within 4 weeks and by the eighteenth week, transgenic hairy roots developed from the surface of callus, with an induction rate of 68.7% (**Figure 3B**, 18 weeks). Fluorescence microscopy analysis revealed strong and uniformly distributed GFP signals in both proliferating callus and induced hairy roots, confirming stable expression of the transgene (**Figure 3C**). The fluorescent tissues showed no signs of chimerism, and GFP expression was particularly evident in root elongation zones, corresponding to regions of active cell division.

### 3.4 Tissue culture-free transformation via high-pressure propagation of woody stems

**Figure 4:**
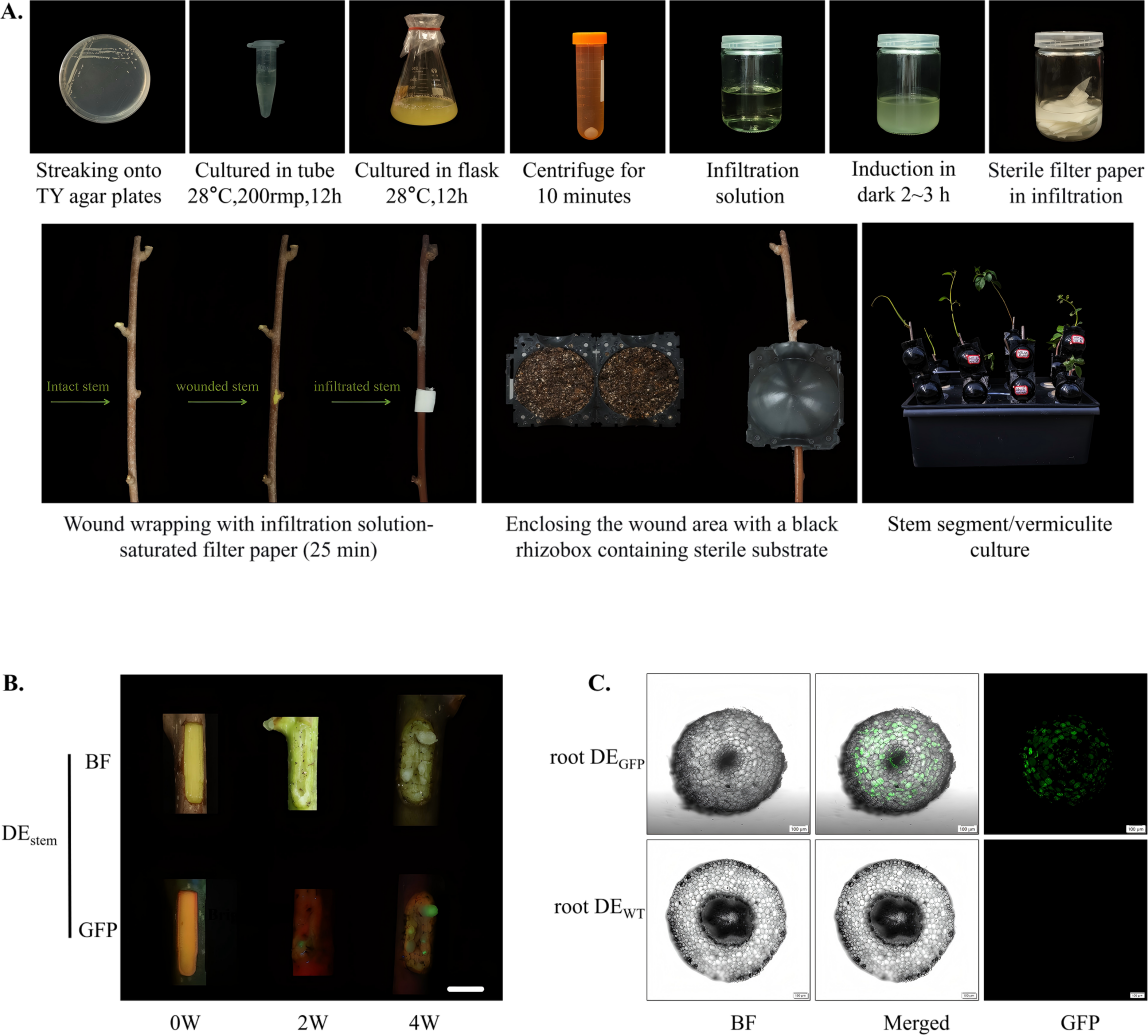
Tissue culture-free transformation of *A. valvata* stems via high-pressure propagation. **(A)** Overview of the High-Pressure Propagation-mediated transformation method using *A. rhizogenes* K599 carrying the 1380-GFP vector. The workflow includes the following sequential steps: streaking onto TY agar plates; cultured in tube (28 °C, 200 rpm, 12 h); cultured in flask (28 °C, 200 rpm, 12 h); centrifugation for 10 minutes; resuspension of *A. rhizogenes* K599 for plant tissue infiltration; induction in dark for 2–3 h; sterile filter paper saturated with infiltration solution; Wound wrapping with infiltration solution-saturated filter paper (25 min): Demonstration of the state comparison among intact stem, wounded stem, and infiltrated stem during the infiltration process; enclosing the wound area with a black rhizobox containing sterile substrate; stem segment/vermiculite culture: Culture system for stem segments after infiltration. **(B)** Phenotypic comparison of the DE_stem_ at different culture stages (0W, 2W, 4W), showing morphological changes under BF and GFP fluorescence. Progressive tissue development and the corresponding variation in fluorescence intensity reflect transgene expression over time. With images captured from different branches at different time points between 0-4W. Scale bar = 1 cm. **(C)** Observation of transgenic roots (root DE_GFP_) under BF, merged, and GFP channels. Distinct green fluorescence signals in transgenic samples confirm successful gene transfer and expression, while no fluorescence is detected in WT (root DE_WT_). Images were captured with a 10× objective.

A reproducible tissue culture-free transformation system was successfully established for *A. valvata* using *A. rhizogenes* K599 under high-pressure propagation conditions. The system exhibited stable performance across independent trials, achieving both high transformation efficiency and reliable gene integration in woody tissues (**Figure 4A**). After 21 days of cultivation, apparent callus formation was observed at the wound sites of inoculated stem (**Figure 4B**). Fluorescence detection using confocal laser scanning microscopy revealed that approximately 50% of the induced callus and hairy roots exhibited bright green fluorescence (**Figure 4C**). The GFP signals were evenly distributed throughout the cytoplasm, confirming stable expression of the reporter gene and the absence of chimerism. The transformation efficiency and GFP verification results demonstrate that this high-pressure propagation-based, tissue culture-free system enables rapid and stable gene introduction into woody stems. Compared with traditional aseptic transformation methods, it markedly shortens the experimental cycle, maintains tissue vitality, and simplifies operation without compromising transformation stability.

## 4 Discussion

This study provides clear evidence of pronounced genotype-dependent variation in GV3101-mediated transformation efficiency between *A. valvata* and *A. chinensis*, offering new insights into germplasm-specific optimization of kiwifruit transformation protocols. Transformation efficiency in *A. chinensis* leaf explants reached 24%, allowing the entire process (from callus induction to complete plant regeneration) to be achieved within approximately 20 weeks. In contrast, *A. valvata* exhibited only 4% efficiency, and its callus failed to develop shoot primordia throughout the culture period. The successful shoot regeneration of *A. chinensis* on induction medium containing 3 mg L^-1^ TZ and 0.5 mg L^-1^ IAA suggests a high sensitivity of *A. chinensis* cells to cytokinin signaling (Su et al., 2011; Yao et al., 2023). Conversely, the developmental arrest observed in *A. valvata* may be linked to impaired hormone signal transduction or epigenetic modifications of key regulatory genes governing shoot differentiation (Li et al., 2011).

The fluorescence-molecular dual verification system established in this study strictly adheres to international quality standards and effectively minimizes false positives. Confocal fluorescence imaging accurately distinguished genuine GFP expression from chlorophyll autofluorescence, confirming the absence of chimeric events. Meanwhile, molecular verification using an optimized CTAB extraction protocol provided high-quality genomic DNA, enabling consistent PCR and sequencing validation. The full concordance between fluorescence observation and molecular results demonstrates the robustness of this dual-verification strategy, making it particularly suitable for identifying true transgenic events in species with low transformation efficiency. Moreover, employing GFP-positive tissues as selection criteria ensures accuracy in subsequent functional studies and provides a standardized reference for extending the method to other reporter genes.

The K599-mediated transformation system developed for *A. valvata* exhibits distinct biological and technical advantages, providing a stable and efficient framework for genetic transformation and root-specific functional studies in this species. Unlike *A. tumefaciens*, the K599 strain harbors the *rol* gene cluster on its Ri plasmid, which autonomously induces hairy root formation without requiring exogenous hormones (Kumar et al., 2025; Liu et al., 2025). This system therefore provides a dedicated platform for investigating root-specific gene functions. Hormonal assays in this study further revealed the developmental plasticity of *A. valvata* callus. In the absence of exogenous hormones, expression of the *rolB* gene exhibited auxin-like activity, activating root primordium initiation (Bose et al., 2022). Conversely, the addition of IAA and thidiazuron (TDZ) appeared to enhance endogenous auxin signaling, suppressing *rol* gene function and maintaining callus in a proliferative state, which is consistent with similar findings in apple hairy root induction (Liu et al., 2024). This system enables functional studies of root-related genes, including those involved in stress tolerance (Lin et al., 2025; Wei et al., 2025). The K599-mediated tissue culture-free transformation system developed here represents a substantial methodological innovation. Conceptually, it shifts from traditional aseptic *in vitro* transformation to directed *in planta* transformation under greenhouse conditions, effectively addressing three long-standing challenges in woody plants such as kiwifruit: strong genotype dependence, high technical barriers, and extended regeneration cycles (Zhang et al., 2025). Conventional GV3101-mediated transformation requires sterile explant preparation and multi-stage regeneration, whereas the high-pressure system achieves direct gene delivery into living stem tissues, bypassing tissue culture entirely. This approach reduced the overall experimental cycle to 4–5 weeks and simplified operation procedures.

The consistent induction of callus and hairy roots at the wound sites of *A. valvata* stems primarily results from the synergistic actions of the K599 Ri plasmid (Xiang et al., 2016). On one hand, genes in the *Vir* region, induced by acetosyringone, initiate T-DNA transfer and integration, ensuring stable gene introduction (e.g., *GFP*). On the other hand, expression of the *rolA/B/C* cluster triggers auxin-like signals that activate host cell dedifferentiation. Unlike immersion-based infection methods that distribute bacteria randomly, this approach targets the cambial cells at wound sites, minimizing non-specific infection and false positives (Liu et al., 2025). From a practical perspective, this system is compatible with CRISPR/Cas9 genome editing and can significantly accelerate molecular breeding in kiwifruit. Recent advances in CRISPR-mediated editing have improved fruit quality traits and enhanced disease resistance through *AcCBL3* modification (Fu et al., 2023; Li et al., 2024). The ability of this system to perform direct *in planta* transformation also allows gene function validation in mature plants, thereby addressing one of the most persistent limitations in perennial fruit crops: long breeding cycles.

## 5 Conclusion

This study systematically developed and optimized multiple *Agrobacterium*-mediated transformation systems for kiwifruit, providing a comprehensive platform adaptable to diverse genotypes. Using GV3101, efficient transformation and complete plant regeneration were achieved in *A. chinensis*, whereas *A. valvata* exhibited limited organogenesis, highlighting genotype-specific constraints. To overcome this, K599-based systems were established, including a hormone-free tissue culture method that induced transgenic hairy roots with 68.7% efficiency and a tissue culture-free high-pressure propagation approach that achieved 50% transgenic efficiency within five weeks. Both systems demonstrated stable GFP expression and confirmed transgene integration by PCR and sequencing. Collectively, these protocols provide rapid, efficient, and reproducible gene delivery pathways for *Actinidia* species, enabling functional genomics, root biology, and gene editing studies. The integration of tissue culture and *in planta* transformation expands the methodological toolkit for woody fruit crops and accelerates molecular breeding in kiwifruit.

## Data Availability

The original contributions presented in the study are included in the article/supplementary material. Further inquiries can be directed to the corresponding author.
